# Supplementary material for: A laboratory-based study to explore the use of honey-impregnated cards to detect chikungunya virus in mosquito saliva
Source: PLoS One. 2021 Apr 1;16(4):e0249471. doi: 10.1371/journal.pone.0249471 (PMC8016228; doi:10.1371/journal.pone.0249471)
Supplement: S1 Table — 50 μL of different dilutions of viral particles were processed for RNA extraction and quantification of RNA copies by qRT-PCR. (PDF) [file pone.0249471.s001.pdf]

A laboratory-based study to explore the use of honey-impregnated cards to detect chikungunya virus in mosquito saliva

Lisa Fourniol,[1] Yoann Madec,[2] Laurence Mousson,[1] Marie Vazeille[1] and Anna-Bella Failloux[1]*

[1]Arboviruses and Insect Vectors Unit, Institut Pasteur, Paris, France
[2]Emerging Diseases Epidemiology Unit, Institut Pasteur, Paris, France

S1 Table. Detection sensitivity of CHIKV RNA copies by qRT-PCR. 50 µL of different dilutions of viral particles were processed for RNA extraction and quantification of RNA copies by qRT-PCR.

Titer of viral stock (pfu/mL)	Quantity of viral particles before qRT-PCR	Quantity of viral RNA copies detected by qRT-PCR
100000000	5×10^6	6.23×10^6
10000000	5×10^5	9.21×10^5
1000000	5×10^4	1.75×10^4
100000	5×10^3	9.44×10^3
10000	5×10^2	8.00×10^2
1000	5×10^1	5.95×10^1
100	5	2.55×10^1
10	1	-
