# Supplementary material for: A laboratory-based study to explore the use of honey-impregnated cards to detect chikungunya virus in mosquito saliva
Source: PLoS One. 2021 Apr 1;16(4):e0249471. doi: 10.1371/journal.pone.0249471 (PMC8016228; doi:10.1371/journal.pone.0249471)
Supplement: S2 Table — Two replicates (R1 and R2) were performed. (PDF) [file pone.0249471.s002.pdf]

A laboratory-based study to explore the use of honey-impregnated cards to detect chikungunya virus in mosquito saliva

Lisa Fourniol,[1] Yoann Madec,[2] Laurence Mousson,[1] Marie Vazeille[1] and Anna-Bella Failloux[1]*

[1]Arboviruses and Insect Vectors Unit, Institut Pasteur, Paris, France
[2]Emerging Diseases Epidemiology Unit, Institut Pasteur, Paris, France

S2 Table. Quantities of RNA copies detected on cards impregnated with honey solutions (10%, 20%, 50%) and examined at different days (1, 2, 3 and 7) after spotting different quantities of CHIKV particles. Two replicates (R1 and R2) were performed.

Honey 10%

Viral RNA deposited on the filter paper	Day 1		Day 2		Day 3		Day 7	
	R1	R2	R1	R2	R1	R2	R1	R2
5 x 10^6	26246	123	4802	15639	1226	2142	118	4471
5 x 10^5	2380	98	83	2925	32	199	1242	93
5 x 10^4	442	128	0	68	25	43	39	0
5 x 10^3	0	0	0	80	0	0	0	0
5 x 10^2	0	0	0	0	0	0	0	21
5 x 10^1	8	11	0	0	145	18	29	0
5	0	31	0	0	0	0	0	58
1	0	0	0	0	8	0	0	0

Honey 20%

Viral RNA deposited on the filter paper	Day 1		Day 2		Day 3		Day 7	
	R1	R2	R1	R2	R1	R2	R1	R2
5 x 10^6	26246,44	123,73	4802,17	15639,06	1226,75	2142,95	118,3	4471,7
5 x 10^5	2380,69	98,09	83,3	2925,59	32,86	199,93	1242,6	93,54
5 x 10^4	442,39	128,09	0	68,59	25,37	43,23	39,42	0
5 x 10^3	0	0	0	80,34	0	0	0	0
5 x 10^2	0	0	0	0	0	0	0	21,04
5 x 10^1	8,45	11,85	0	0	145,69	18,3	29,71	0
5	0	31,99	0	0	0	0	0	58,95
1	0	0	0	0	8,26	0	0	0

Honey 50%

Viral RNA deposited on the filter paper	Day 1		Day 2		Day 3		Day 7	
	R1	R2	R1	R2	R1	R2	R1	R2
5×10^6	186415	120008	111794	454	16003	0	26964	19266
5×10^5	67300	8113	13835	0	1376	0	1591	1910
5×10^4	2065	1091	1054	0	178	27	90	0
5×10^3	302	124	225	55	29	0	45	105
5×10^2	0	0	0	1288	0	0	0	0
5×10^1	0	45	0	0	0	0	0	0
5	734	0	0	29	0	0	0	0
1	0	0	0	0	0	50	0	2
