# Supplementary material for: A laboratory-based study to explore the use of honey-impregnated cards to detect chikungunya virus in mosquito saliva
Source: PLoS One. 2021 Apr 1;16(4):e0249471. doi: 10.1371/journal.pone.0249471 (PMC8016228; doi:10.1371/journal.pone.0249471)
Supplement: S3 Table — (PDF) [file pone.0249471.s003.pdf]

A laboratory-based study to explore the use of honey-impregnated cards to detect chikungunya virus in mosquito saliva

Lisa Fourniol,[1] Yoann Madec,[2] Laurence Mousson,[1] Marie Vazeille[1] and Anna-Bella Failloux[1]*

[1]Arboviruses and Insect Vectors Unit, Institut Pasteur, Paris, France
[2]Emerging Diseases Epidemiology Unit, Institut Pasteur, Paris, France

S3 Table. Comparison of transmission efficiencies between mosquito species, day post-exposure and treatments (logistic regression model).

	N	Transmission efficiency (N)	Crude OR (95% CI)	p
**Species**				
Aedes aegypti	263	55.5 (146)	1	0.25
Aedes albopictus	182	61.0 (111)	1.25 (0.85 – 1.84)	
**Day post-exposure**				
3	237	43.5 (103)	1	<0.0001
7	208	74.0 (154)	3.71 (2.48 – 5.55)	
**Treatments**				
Card unchanged	110	50.0 (55)	1	0.0007
Card changed	99	46.5 (46)	0.90 (0.50 – 1.49)	
Saliva collection	236	66.1 (156)	1.95 (1.23 – 3.09)	

OR: Odd Ratio, is a measure of association which compares the odds of infection of mosquitoes exposed to the infectious blood meal to the odds of infection of unexposed mosquitoes.
