# Supplementary material for: A laboratory-based study to explore the use of honey-impregnated cards to detect chikungunya virus in mosquito saliva
Source: PLoS One. 2021 Apr 1;16(4):e0249471. doi: 10.1371/journal.pone.0249471 (PMC8016228; doi:10.1371/journal.pone.0249471)
Supplement: Attachment — Submitted filename: MS_Fourniol_Answers to Reviewers-2_17 03 2021.doc [file pone.0249471.s005.doc]

| **Arboviruses and Insect Vectors** |  |  |
| --- | --- | --- |

Dear Editor,

We would like to thank the two reviewers for their efforts in improving our manuscript by Fourniol et al. Please, find below point-by-point answers to the reviewers, indicating which changes have been made, and where they have been inserted in the text (highlighted in yellow in the revised version). We remain at your disposal to answer to any other comments.

Sincerely yours,

**Prof. Anna-Bella FAILLOUX**

Institut Pasteur, Department of Virology

Arboviruses and Insect Vectors Unit

25 rue du Dr Roux,75724 Paris cedex 15

**Answers to Reviewer #1:**

***1) Initial question - #2a - Were any uninfected controls to help gauge the false positive rate?***

***Answer - To answer properly to this question, we need more details
Follow-up: I will clarify my comment: As a control in experimental infections, it’s worthwhile to include a mock infection treatment group - mosquitoes that were not exposed to CHIKV-infected blood, and were instead fed naïve blood, or blood plus sterile culture media. Mosquitoes from this treatment would then be allowed to salivate on the honey-impregnated cards in the same manner as CHIKV-exposed mosquitoes in order to assess the accuracy of the viral detection/quantification methodology. If a similar control group was included in the experiments, the results should be discussed in the paper. If no control group was included that should be specified in the methodology.***

We understand but we did not expose mosquitoes to a non-infectious blood meal.

We have inserted this statement in line 128: “Non-infected mosquitoes (exposed to non-infectious blood or culture media) were not tested”.

***1) Initial question - #4 - Lines 60-62 “Honey-coated cards have been used successfully to detect arbovirus circulation (namely, West Nile virus, Ross River virus, Barmah Forest virus, Japanese encephalitis virus and CHIKV) (16-19).” – what is different between this historical CHIKV study and the current study?
In Hall-Mendelin et al. (2010; PMID: 20534559),
Answer***

***- the design of experiments is different:***

***-Mosquitoes used are from a different region***

***-CHIKV strain was isolated from a patient in Australia and viral stocks were obtained after three passages on vertebrate (Vero) cells***

***-12 days after experimental infection, mosquitoes were exposed to cards for 48 h
The authors found 75% (21/28) of mosquitoes that had expectorated the virus on honey-impregnated filters compared to 52% (14/27) of mosquitoes that have virus detected in saliva collected using a capillary tube. There was no significxant difference between the two methods.***

***Follow-up: These details need to be included somewhere in the introduction or discussion.***

As suggested, we have added in lines 261-263: “Its gives results comparable to those obtained using the standard forced salivation technique of mosquitoes (16)”.

**Answers to Reviewer #2**

***1) The authors should incorporate into the discussion few sentences to explain how they work complete the previous work from Hall-Mendelin et al., 2010 (doi: 10.1073/pnas.1002040107). This will help the readers to better understand the novelty of this paper.***

We have added in lines 261-263: “Its gives results comparable to those obtained using the standard forced salivation technique of mosquitoes (16)”.

***2) While the authors have modified the table 1 to incorporate percentages, many of percent symboles are still missing. Could the authors carefully correct this point?***

Thanks for noticing it. It has been corrected.
